# Molecular and Immobilized Tripodal Phosphine Ligands and Their Trinuclear Palladium Complexes

**DOI:** 10.3390/molecules30071616

**Published:** 2025-04-04

**Authors:** Maxwell R. Kimball, Kyle J. Cluff, Nattamai Bhuvanesh, Janet Blümel

**Affiliations:** Department of Chemistry, Texas A&M University, College Station, TX 77842-3012, USAnbhuv@chem.tamu.edu (N.B.)

**Keywords:** tripodal phosphines, immobilized phosphines, trinuclear Pd complexes, virtual couplings, immobilized Pd complexes, *cis*- and *trans*-coordinated Pd complexes, immobilized Cu complex, silica as support, ^31^P solid-state NMR, alkoxysilanes

## Abstract

The synthesis and characterization of the tripodal phosphines RSi(CH_2_CH_2_PPh_2_)_3_ (R = Me, OMe, OEt) (**1**–**3**) is described. The ^1^H NMR spectra of all phosphines display virtual coupling patterns. The ligands form the corresponding trinuclear Pd complexes [RSi(CH_2_CH_2_PPh_2_)_3_]_2_(PdCl_2_)_3_ (**4**–**6**) with three PdCl_2_ moieties sandwiched between two tripodal ligands. The complexes **4**, **5**, and **7** (R = OH) have been analyzed by single crystal X-ray diffraction. The coordination at the Pd center is square planar with the phosphine groups occupying *trans* positions. The ^31^P{^1^H} MAS NMR spectra of polycrystalline **1** are in accordance with the packing motif of the molecules in the unit cell. The tripodal ligand **3** has successfully been immobilized on silica as **3i**. It coordinates PdCl_2_ on the surface, as demonstrated by ^31^P{^1^H} MAS NMR. Hereby, the *cis* coordination is prevalent when **3i** has maximal surface coverage. At low surface coverage, one tripodal linker can accommodate *trans* coordination at the metal center. A surface-bound trinuclear Pd complex has been generated, as well as a heterobimetallic Pd/Cu complex. All surface species have been characterized by ^31^P{^1^H} MAS NMR.

## 1. Introduction

### General Introduction

**Figure 1:**
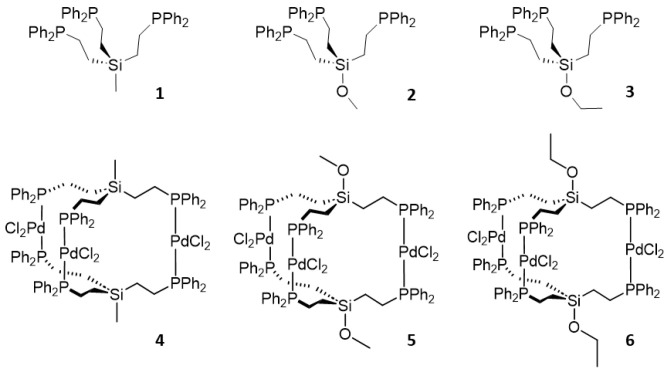
Tripodal phosphine ligands **1**–**3** and the corresponding trinuclear Pd complexes **4**–**6**.

One of our main research interests is immobilizing molecular catalysts on solid supports to allow for easy separation from the reaction mixtures and recycling [1]. Silica has been mostly chosen as the support material because it is inexpensive and available in a variety of pore and particle sizes. It is temperature stable and mechanically robust and can be separated by settling. It retains the immobilized catalyst even in cases when metal nanoparticles form [2,3,4]. Bifunctional phosphines have proven to be favorable linkers because they can coordinate metal complexes and irreversibly bind to silica surfaces. The latter can be accomplished by creating phosphonium salts on the surface [2,4,5], however, the more straightforward way of immobilization is via the reaction of alkoxysilyl groups with surface silanol and siloxane groups [6]. Catalysts immobilized with mono-, di-, and triphosphine linkers [1,3] incorporating ethoxysilyl groups could be recycled many times in a batchwise manner [3]. In general, chelating phosphine ligands increase the lifetimes of the immobilized catalysts because leaching is diminished, as demonstrated by HRMAS NMR spectroscopy earlier [7,8,9]. Tripodal phosphine linkers, in particular, offer the potential to coordinate two different metals with one linker [10], as desirable, for example, for the Sonogashira reaction [9]. Tripodal phosphine linkers have successfully been applied for immobilizing Wilkinson-type rhodium hydrogenation catalysts [2,3]. However, one last challenge remains. After several catalytic runs, most often metal nanoparticles form because of the mobility of the phosphine linkers in the presence of a solvent that allows for contact of the metal centers [2,3]. This process occurs especially when linkers with long, flexible methylene chains of up to 11 CH_2_ segments are used [2,3]. In order to suppress the nanoparticle formation, while retaining the favorable features of the chelate phosphines, we opted for tripodal phosphine linkers with shorter methylene chains incorporating alkoxy groups for immobilization on silica (Figure 1). Unfortunately, the tripodal phosphine congeners with only one methylene group tend to decompose when immobilized on silica [11,12].

**Figure 2:**
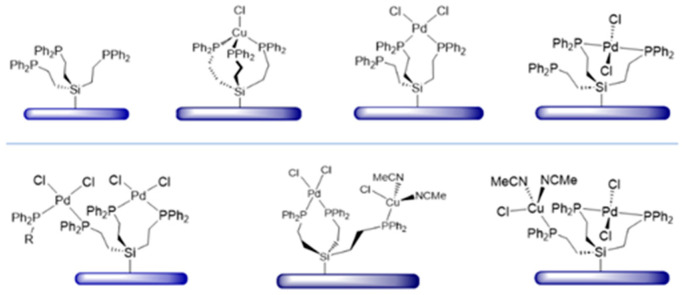
Immobilized tripodal phosphine ligand **3i** and the corresponding trinuclear Pd, Cu, and Pd/Cu complexes.

However, the tripodal ligand **3** with ethylene groups separating the ethoxysilyl and phosphine groups, can be bound to silica as **3i** without decomposition (Figure 2). The tripod structure remains intact, as demonstrated by solid-state NMR spectroscopy.

In this contribution, we describe the synthesis and characterization of the previously unused tripodal phosphine linkers of the type RSi(CH_2_CH_2_PPh_2_)_3_ (R = Me, OMe, OEt) (**1**–**3**) and their trinuclear Pd complexes [RSi(CH_2_CH_2_PPh_2_)_3_]_2_(PdCl_2_)_3_ (**4**–**6**) (Figure 1). The complexes **4**, **5**, and **7** (R = OH) have been analyzed by single crystal X-ray diffraction. It has been demonstrated by ^31^P{^1^H} MAS NMR that the tripodal ligand **3** can successfully be immobilized on silica as **3i** and that PdCl_2_ and CuCl can be bound to it. Diverse mono- and heterodinuclear surface-bound complexes could be identified (Figure 2).

## 2. Results and Discussion

### 2.1. Tripodal Phosphine Ligands

The tripodal phosphine ligands **1**–**3** (Figure 1) have been synthesized by radical-initiated hydrophosphination of the corresponding trivinylsilanes with HPPh_2_. The reactions could be initiated by UV irradiation, as described previously for **1** [13], and the tripodal phosphine EtOSi(CH_2_PPh_2_)_3_ [11,12]. Alternatively, AIBN (azobisisobutyronitrile) can function as a radical initiator, as described for the synthesis of tripodal phosphine ligands with long methylene chains incorporating ethoxysilyl groups [2,3]. The tripodal phosphines **1**–**3** were obtained as colorless solids in yields higher than 84%.

The ^31^P{^1^H} NMR signals of the tripodal ligands **1**–**3** are found within the narrow chemical shift range from −9.4 to −9.9 ppm (Figure S1). There is only one clean singlet for each tripodal ligand, indicating that all PPh_2_ groups are chemically equivalent.

**Figure 3:**
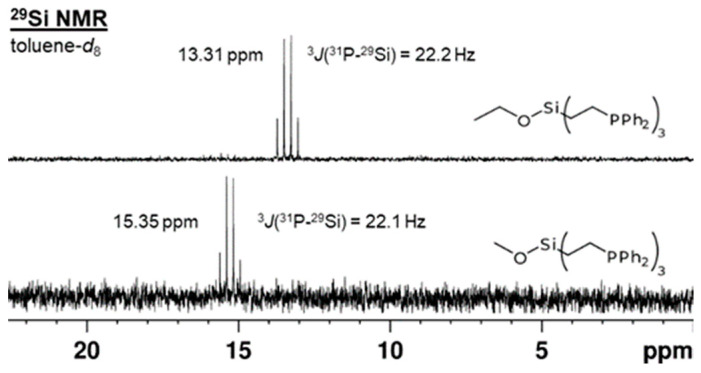
^29^Si{^1^H} NMR spectra of **2** (**bottom**) and **3** (**top**) in toluene-*d*_8_.

Direct proof for the presence of three equivalent phosphorus atoms in the tripodal ligands **1**–**3** can be derived from the ^29^Si{^1^H} NMR spectra (Figure 3). The ^29^Si{^1^H} signals are 1:3:3:1 quartets with ^3^*J*(^31^P-^29^Si) coupling constants of 21.2 Hz (**1**), 22.1 Hz (**2**), and 22.2 Hz (**3**), in accordance with values for similar systems that range from 12.1–27.8 Hz [14,15,16]. The *δ*(^29^Si) of **1**–**3** lie within the expected chemical shift range for tetraalkyl- and alkoxytrialkylsilanes from 0 to 20 ppm [11,12].

The ^1^H NMR spectra of all tripodal ligands show virtual couplings for the signals of the diastereotopic ethylene protons (Figures S2–S5). The signal shapes are textbook examples of virtual couplings [1,11,17,18]. The shapes and the spans of the signals, defined as the Hz distances between the outer lines, do not change when the strength of the external magnetic field is increased from 400 to 500 MHz. For example, the methylene signals of **3** at 1.87 ppm and 0.67 ppm retain the distances of 17.7 Hz and 27.0 Hz between their outer lines (Figure S5). When the solvent is changed from CDCl_3_ to C_6_D_6_, a slight change of the distances to 17.3 Hz and 27.2 Hz occurs.

In addition to the ^1^H NMR spectra, ^13^C{^1^H} NMR (Figures S6 and S7) confirms the presence of the methyl, methoxy, and ethoxy groups bound to Si in **1**–**3**. The phenyl carbon signals have the expected chemical shifts and display the characteristic ^1–3^*J*(^31^P-^13^C) couplings of the *ipso*, *ortho*, and *meta* carbon nuclei with ^31^P that have been observed for tripodal diphenylalkylphosphines previously [2,3,11].

### 2.2. Trinuclear Palladium Complexes

**Figure 4:**
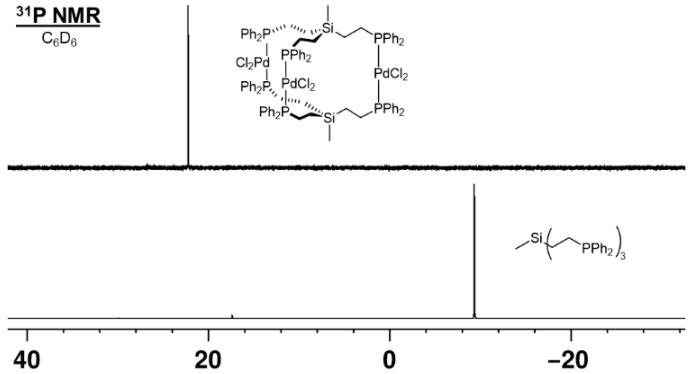
^31^P{^1^H} NMR spectrum of **1** and corresponding PdCl_2_ complex **4** in C_6_D_6_.

When the tripodal phosphines **1**–**3** are reacted with (PhCN)_2_PdCl_2_ the complexes **4**–**6** result as polycrystalline solids (Figure 1). The yields are respectable but not quantitative because of the intrinsic potential of tripodal ligands to form coordination polymers in solution. Consequently, the ^31^P{^1^H} NMR signals undergo a characteristic downfield shift that indicates the coordination of the phosphines to the metal centers (Figure S1 and Figure 4). All phosphorus nuclei are chemically equivalent due to the symmetry of the complexes and therefore, their signals show no couplings.

**Figure 5:**
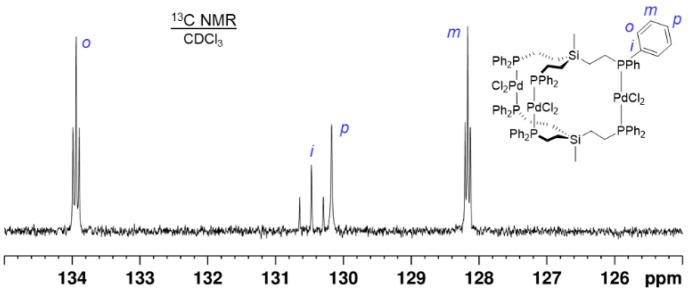
^13^C{^1^H} NMR spectrum of **4** in CDCl_3_. The *ipso*, *ortho*, and *meta* carbon signals show virtual couplings.

However, the ^13^C{^1^H} NMR spectrum of **4**, for example, shows virtual couplings for the *ipso*, *ortho*, and *meta* carbons (Figure 5 and Figure S8) [1,11,17,18]. This means that different ^31^P nuclei communicate with each other and the ^13^C{^1^H} NMR signals are split into triplets by seemingly identical phosphorus coupling partners [17,18]. In principle, the *J*(^31^P-^13^C) couplings could be propagated along the pathway over the Si atom. However, in this case, more complex coupling patterns would be expected and the ^13^C{^1^H} NMR signal of the carbon bound to Si should also be split into a multiplet. This is not the case (Figure S8) but the alkyl carbon bound to ^31^P features a virtual triplet. Therefore, it can be concluded that this triplet and the virtual couplings to the aryl carbon nuclei are due to the coupling pathway over the Pd center.

### 2.3. X-Ray Crystallography

**Figure 6:**
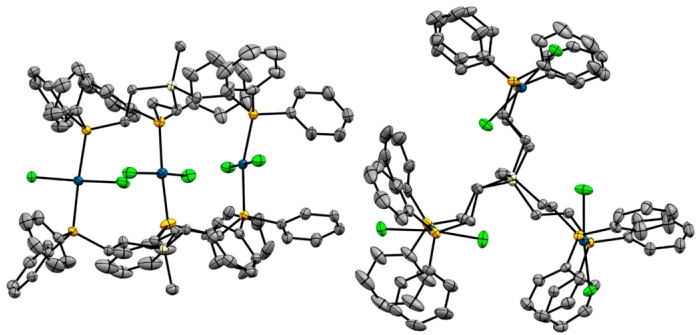
Single crystal X-ray structure of one molecule of **4**. View from the side (**left**) and along the Si···Si axis (**right**). Hydrogen atoms, solvent molecules, and disordered phenyl rings are omitted for clarity.

**Figure 7:**
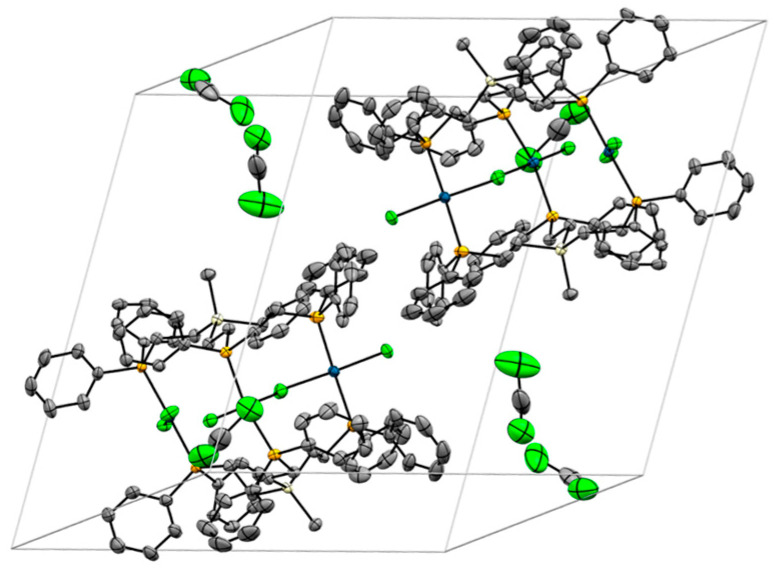
Unit cell of the single crystal X-ray structure of **4**, incorporating six solvent molecules (DCM). Hydrogen atoms and disordered phenyl rings are omitted for clarity.

**Figure 8:**
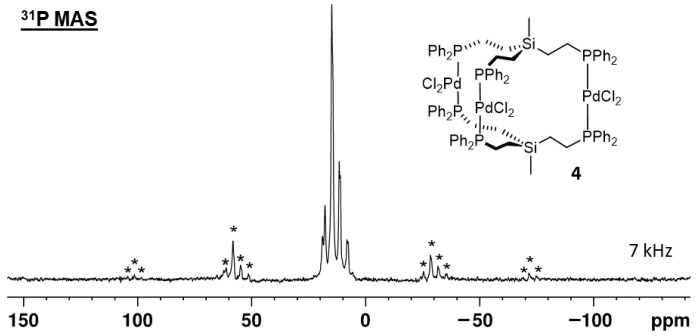
^31^P{^1^H} MAS NMR spectrum of polycrystalline **4**, rotated with a spinning frequency of 7 kHz. Asterisks denote rotational sidebands.

**Figure 9:**
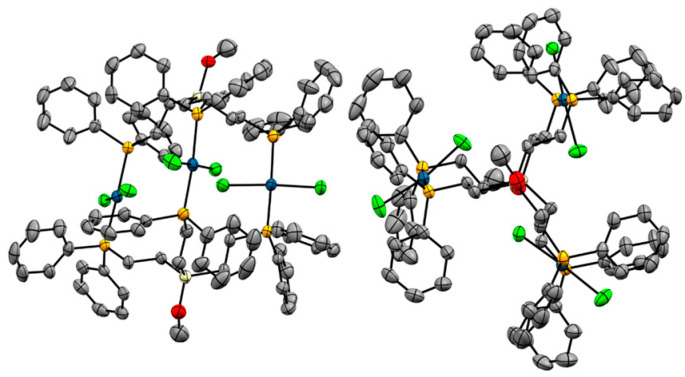
Single crystal X-ray structure of one molecule of **5a**. View from the side (**left**) and along the Si···Si axis (**right**). Hydrogen atoms and disordered solvent molecules are omitted for clarity.

**Figure 10:**
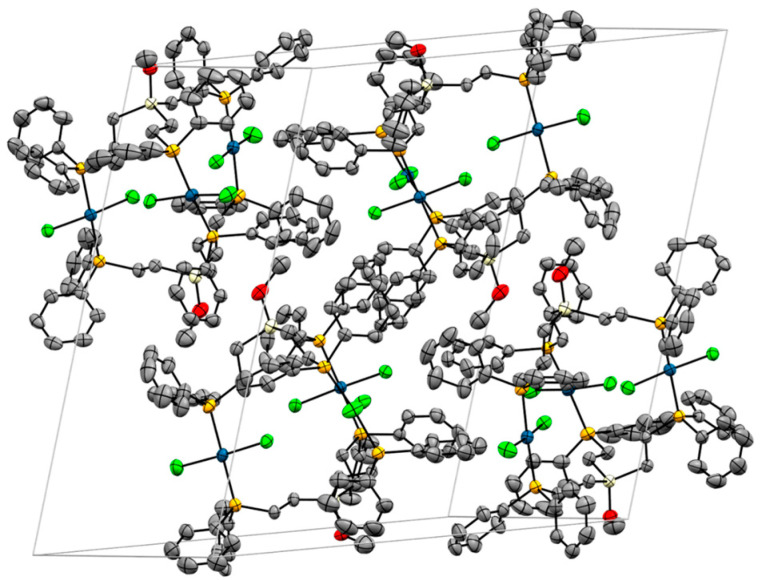
Unit cell of **5a**. Hydrogen atoms and disordered solvent molecules are omitted for clarity.

**Figure 11:**
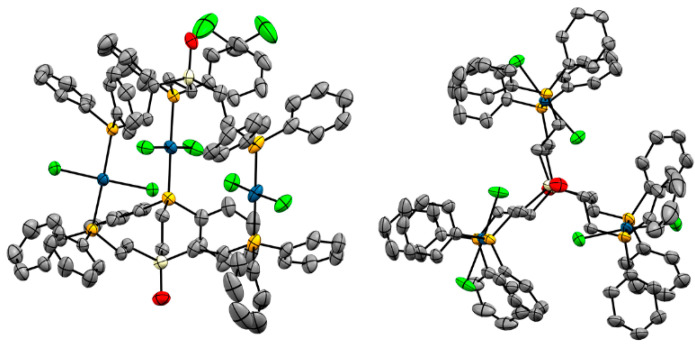
Single crystal X-ray structure of one molecule of **7** with one included dichloromethane molecule. View from the side (**left**) and along the Si···Si axis (**right**). Hydrogen atoms are omitted for clarity.

The trinuclear Pd complexes **4** and **5** could be crystallized from a solvent mixture of DCM with ether and single crystal X-ray diffraction confirmed the structures depicted in Figure 1. Unfortunately, high-quality crystals of **6** could not be obtained because the ethoxysilyl group was prone to alkoxy exchange and hydrolysis. Attempts to crystallize **6** in the presence of MeOH led to alkoxy exchange and crystals of **5**. Single crystals of **5** obtained from MeOH (**5a**) show significant structural differences compared to the crystals of **5** obtained from DCM/ether mixtures (**5b**). Traces of water in the solvent used for crystallizing **6** led to the X-ray structure of the corresponding silanol **7** (R = OH). All data and measurement details for X-ray diffraction are reported in the Supplementary Materials and in ref. [19]. The single crystal structures and unit cells of **4**, **5**, and **7**, as well as solid-state NMR spectra of polycrystalline **4** are displayed in Figure 6, Figure 7, Figure 8, Figure 9, Figure 10 and Figure 11, Figures S9 and S10.

The structure of **4** and its unit cell are displayed in Figure 6 and Figure 7. Three PdCl_2_ units are sandwiched between two tripodal phosphine ligands **1**. The phosphine groups occupy the *trans* positions and all P–Pd–P axes are nearly parallel.

Interestingly, the view from the top of the complex along the Si···Si axis reveals that the ethylene groups of the tripodal ligands are nearly eclipsed with very small dihedral angles (Figure 6). Even the phenyl groups are parallel. The orientation of the Cl atoms, however, appears to be random and dominated by the packing motif. Under the conditions of the X-ray diffraction measurement the Cl–Pd–Cl moieties of **4** do not rotate although the available space would allow for this [20].

The unit cell of **4** is composed of two molecules. The remaining voids are filled with six DCM molecules. Regarding the arrangement of the complexes within the unit cell, there are 12 magnetically inequivalent phosphorus nuclei. However, the two complex molecules have a similar orientation in space, and, therefore, pairs of two phosphorus nuclei in analogous positions are expected to yield similar chemical shifts in the ^31^P{^1^H} MAS NMR spectrum of solid **4**. To test this hypothesis, we recorded the ^31^P{^1^H} MAS spectra of polycrystalline **4** at different rotational speeds (Figure 8 and Figure S11). As expected, the spectra show multiple, partly overlapping, signals with at least six isotropic lines. The chemical shift range of the isotropic lines corresponds to the *δ*(^31^P) of alkyldiphenylphosphines *trans*-coordinated to PdCl_2_ [7,8,9].

Figure 9 displays the structure of **5**, viewed from the side and along the Si···Si axis. The structural features are very similar to those discussed above for **4**, although the alignment of the ligand ethylene groups is not quite as precisely eclipsed as in **4**. The biggest difference is visible in the unit cell of **5** (Figure 10). Four trinuclear complexes are densely packed in the unit cell, with disordered solvent molecules filling the voids.

The structure of one molecule of **7** resembles more closely the one of **4** (Figure 11). However, the unit cell is different and shows a very complex packing motif (Figure S12).

Interestingly, the trinuclear sandwich motif we found for **4**–**7** has only been described previously for two Pd complexes [21] and one Ni complex [22]. In the case of PdI_2_ and PdCl_2_, the tripodal ligands were O=P(CH_2_CH_2_PPh_2_)_3_ and O=P(CH_2_CH_2_P(S)Ph_2_)_3_, respectively. The ligand in the trinuclear NiCl_2_ complex was CH_3_C(CH_2_CH_2_PPh_2_)_3_. In the following, we include these complexes in our discussion of the X-ray data.

The unit cell and molecular structure of **4** and the complex **Pd_3_I_6_(pOp_3_)_2_** [21] are nearly identical, with close to perfect eclipse of the methylene and phenyl groups as observed in Figure 6.

In the square planar complexes with *trans* coordination of the ligands L, ideally the angles L–M–L should be 180°. However, the values provided in Table 1 show that there is significant scatter, sometimes within the same complex. For example, the structure of **5**, derived from Et_2_O/DCM crystallization (**5b**), shows one significantly smaller L–M–L value of 165.45 (3)° as compared to 176.33 (3)° and 176.45 (3)°. In other words, this complex has an indentation on one side. The most symmetric shape is reported for the complex with an additional S atom that extends the tripodal ligand in **Pd_3_Cl_6_(pOp_3_S_3_)_2_ [21]**.

The X–M–X angles of the PdCl_2_ complexes are closer to the ideal 180° than the PdI_2_ or NiCl_2_ complexes (Table 2). These differences are probably due to the smaller atomic radius of Ni(II) and the larger radius of iodide in the complex Pd_3_I_6_(pOp_3_)_2_, leading to interactions between the halide and phenyl and methylene groups (Table 2). These interactions are minimized by the additional sulfur atom of the complex Pd_3_Cl_3_(pOp_3_S_3_)_2_, which possesses X–M–X angles of 179.5 (1)°, which are closest to the ideal 180° [21].

With the exception of the complex Pd_3_Cl_3_(pOp_3_S_3_)_2_, the ethylene groups in all tripodal ligands in the sandwich complexes in Table 3 are eclipsed. The L–Center–Center–L dihedral angles amount to less than 12.7° for all complexes, except for Pd_3_Cl_3_(pOp_3_S_3_)_2_ (50.68°). The eclipsed ethylene groups in the complex **4**, **5a**, and **7** are visible, for example, in the views along the Si···Si axes (Figure 6, Figure 9, and Figure 11).

The distances between the central atoms of the ligands vary within a large range from 4.85 (9) to 8.530 (2) Å (Table 4). These distances correlate inversely with the distances between the metal centers. For example, complex **7** has the largest distance between the centers, corresponding to relatively small M-M distances. This is understandable, as elongating the “balloons” at the top and bottom will result in more svelt molecules with reduced circumference in the middle due to the flexible nature of the tripodal ligands. Overall, Pd_3_Cl_3_(pOp_3_S_3_)_2_ has the longest metal-metal distances, however, the extra sulfur atom between the metal and center has to be acknowledged. Interestingly, for the center-center and metal-metal distances, the nature of the metal atom, whether it is Pd or Ni, does not seem to play a crucial role (Table 4).

### 2.4. Immobilization on Silica

One of the beneficial features of immobilized tripodal ligands is that coordination polymers can no longer occur. With low surface coverages, coordination of the metal by a second ligand is no longer possible because of the large, fixed distance between the ligands. In contrast to adsorbed species, covalently silica-bound ligands are not mobile on the surface. Furthermore, once the linker containing the alkoxysilyl group is bound to silica, it is no longer water-sensitive. In contrast to **6,** it incorporates a Si-O-C linkage that is easily hydrolyzed to a silanol group to form **7**, once a siloxane (Si-O-Si) group is formed by covalent binding to the silica surface, the linker is no longer prone to hydrolysis. In order to remove a phosphine linker from the silica surface, harsh conditions are needed, as described earlier [23,24].

**Figure 12:**
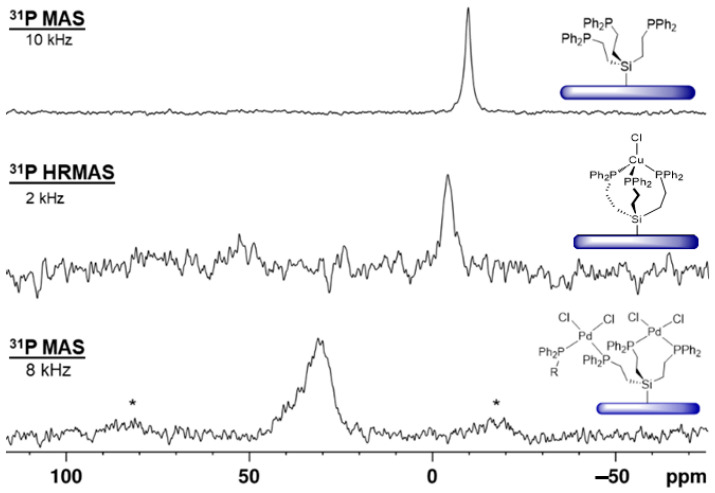
^31^P{^1^H} MAS NMR spectrum of the immobilized tripodal linker **3i** (**top**), ^31^P HRMAS of the immobilized CuCl complex (**middle**), recorded in the presence of acetonitrile, and ^31^P{^1^H} MAS spectrum of the *cis*-coordinated PdCl_2_ complex (**bottom**) at the indicated rotational speeds. Asterisks denote rotational sidebands.

The tripodal phosphine ligand **3** has successfully been immobilized on silica as **3i** according to optimized procedures [1,6,23,24]. The ^29^Si CP/MAS (cross-polarization in combination with magic angle spinning) shows the corresponding signal at 10 ppm, in accordance with the chemical shift of ^29^Si{^1^H} NMR signals of silica-tethered trialkylethoxysilanes [3]. In the ^31^P{^1^H} MAS spectrum a signal at –10 ppm is found (Figure 12, top), in agreement with the *δ*(^31^P) value of the molecular ligand **3** in solution (−9.39 ppm).

When the ligand **3i**, immobilized on silica with maximal surface coverage [23,24], is treated with (MeCN)_2_PdCl_2_ all phosphine groups are coordinated to the metal center. The signal is broad with a characteristic CSA (chemical shift anisotropy) that manifests in large first-order rotational sidebands even at 8 kHz rotational frequency (Figure 12, bottom) [9]. The chemical shift of about 30 ppm indicates that the phosphines are mostly *cis*-coordinated to the metal center [7,8,9]. Since the surface coverage is dense, the Pd complex can span different neighboring tripodal ligands as depicted in Figure 12.

The immobilized ligand **3i** is not limited to coordinating Pd. When 1.5 equivalents of CuCl are added, the surface-bound triphosphine copper chloride complex depicted in Figure 12 (middle) is obtained. The chemical shift of about −5 ppm for the ^31^P{^1^H} HRMAS (high-resolution magic angle spinning) [7,8,9] NMR signal corresponds well to the value reported earlier [9,25].

**Figure 13:**
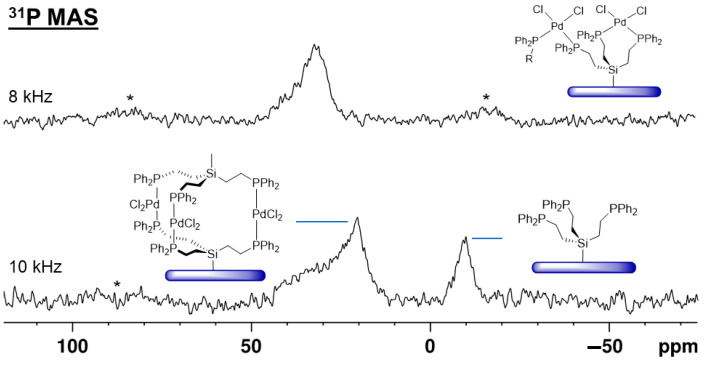
^31^P{^1^H} MAS spectrum of the Pd complex immobilized with maximal surface coverage of **3i** (**top**), and the spectrum obtained after treatment with **1** (**bottom**) at the indicated rotational speeds. The asterisks denote rotational sidebands.

Interestingly, treating the Pd complex, *cis*-coordinated by **3i** (Figure 13, top), with the tripodal ligand **1**, the signal for the uncoordinated surface-bound phosphine **3i** reappears (Figure 13, bottom). This means that ligand **1** replaces the phosphine groups of **3i** at the Pd center. Furthermore, the *cis*-coordinated Pd complex mostly turns into the surface-bound sandwich complex shown in Figure 13. This immobilized complex corresponds to the molecular complex **4**, with *trans* coordination of the phosphine ligands and a ^31^P chemical shift of about 22 ppm. These results are in accordance with earlier studies that suggested that the PdCl_2_ moiety is able to move from one surface-bound phosphine ligand to a more favorable one in the presence of a solvent [7,8,9]. In the presented scenario, *trans* coordination at the Pd center is clearly preferred and is the driving force for the formation of the immobilized trinuclear Pd complex.

**Figure 14:**
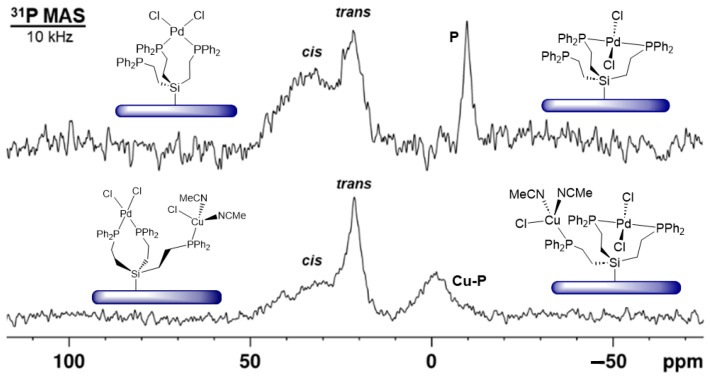
^31^P{^1^H} MAS NMR spectra (10 kHz) of immobilized tripodal linker **3i** (24% surface coverage) *cis*- and *trans*-coordinated to PdCl_2_ (**top**), and the same material after addition of the Cu component (**bottom**). **P** stands for uncoordinated Ph_2_P groups, and **Cu-P** indicates the signal of phosphine groups coordinated to Cu.

When the ligand **3i** is immobilized with only 24% of the maximal surface coverage and treated with one equivalent of (MeCN)_2_PdCl_2_, the ^31^P{^1^H} MAS signal of the uncoordinated ligand is still visible at about –10 ppm (Figure 14, top). This means that in the solid state, there is no intra- or intermolecular rapid ligand exchange, as in the presence of a solvent [7,8,9]. Furthermore, the signal of *cis*-coordinated phosphines appears at about 30 ppm, and in addition, a resonance for *trans*-coordinated phosphine groups (Figure 14, top) at ca. 22 ppm. Since ligand **3i** is dilute on the surface in this case, the tripodal ligand obviously has a large enough span to accommodate the intramolecular *trans* coordination of Pd.

One of our key interests is creating an efficient immobilized Sonogashira Pd/Cu catalyst system that has been described for coupling iodobenzene and phenylacetylene using mono- and bidentate phosphine linkers previously [7,8,9]. The tripodal ligand **3i** allows for both metals to be bound with one linker molecule. This is favorable because the Pd component does not have to migrate to the Cu complex prior to the onset of catalysis [9]. Starting with the ligand **3i** that has been tethered to silica with a low surface coverage of 24% and then treated with PdCl_2_ (Figure 14, top), the Cu complex can be bound by the remaining uncoordinated phosphine group. Accordingly, after treatment with (MeCN)_3_CuCl, the signal for uncoordinated phosphine groups has disappeared, while the resonance for Cu-bound phosphines emerges at about –3.5 ppm (Figure 14, bottom). There is an obvious preference for the *trans* coordination at Pd to persist, while the signal for the *cis*-coordinated phosphines is diminished in intensity. Preliminary experiments show that the combined immobilization of the Pd and Cu components leads to an active Sonogashira catalyst and detailed catalysis studies using this system will be undertaken to compare it with previously reported catalysis results [8,9].

Finally, it should be pointed out that the ^31^P{^1^H} MAS signals obtained for the phosphine groups coordinated to the Pd center are not identical to the phosphine oxide signal. When the immobilized ligand **3i** is reacted with aqueous H_2_O_2_/acetone, all phosphines are turned into phosphine oxides [26]. The signal appears at 38 ppm, downfield-shifted enough to distinguish it from the resonances of Pd-coordinated phosphines (Figure S13).

## 3. Conclusions

Three tripodal phosphine ligands incorporating ethylene spacers and silyl groups have been synthesized and characterized by ^1^H, ^13^C{^1^H}, ^29^Si{^1^H}, and ^31^P{^1^H} NMR spectroscopy (**1**–**3**). All phosphines displayed virtual couplings for the ethylene proton signals and the ^29^Si resonances were split into quartets due to the couplings to three ^31^P nuclei. The trinuclear Pd complexes **4**–**6** have been synthesized with tripodal ligands and characterized by single-crystal X-ray diffraction and multinuclear NMR spectroscopy. The structures display *trans* coordination for all three Pd centers that are sandwiched between two tripodal ligands. The ethylene groups of the tripodal ligands are eclipsed.

In summary, the described NMR spectroscopic and X-ray diffraction results expand the general understanding of molecular and surface-bound metal complexes with tripodal phosphine ligands. Pd- and Cu-containing complexes could be generated on a silica surface and the foundation for the next generation of immobilized Sonogashira catalyst systems with two different metals bound to one tripodal ligand has been established [9,10]. Preliminary results indicate that the presented immobilized Pd/Cu system, in combination with phenylacetylene and iodobenzene, is an active Sonogashira catalyst under the conditions described previously [7,8,9]. In future projects, the catalytic activities of these systems will be explored in detail, optimized, and compared to those of Sonogashira catalysts with separate Pd and Cu components on a silica surface [7,8,9].

## 4. Experimental Section

### 4.1. General Information

All reactions were performed under an inert atmosphere unless specified otherwise. The solvents were dried in a conventional solvent purification apparatus and subsequently kept under purified nitrogen, with the exception of methanol. Methanol was degassed by 3 freeze-pump-thaw cycles and kept over 4 Å molecular sieves for 3 days before use. The melting points and ranges were obtained with an Optimelt melting point apparatus using sealed capillaries. The compounds started melting at the given lower values and reached the clear points at the high values. The silica (average pore diameter 40 Å, particle size 0.06−0.2 mm, specific surface area 750 m^2^/g) was acquired from SiliCycle and dried at 300 °C and 0.1 Torr for 3 days before use in order to allow for the ethoxysilane to bind to the surface [27]. The maximal surface coverage (100%) was determined by offering a weighed excess of **3** in the immobilization step. The original amount of **3** minus the residual **3** found in the supernatant and washings after the immobilization corresponds to the surface-bound **3i** with maximal surface coverage.

### 4.2. NMR Spectroscopy

The ^1^H, ^13^C{^1^H}, ^29^Si{^1^H}, and ^31^P{^1^H} solution NMR spectra were recorded on a Varian VnmrS 500, Varian Inova 500, or Bruker Avance Neo 400 NMR spectrometer. ^1^H and ^13^C{^1^H} NMR spectra were internally referenced to solvent signals. The ^29^Si{^1^H} NMR spectra were externally referenced to hexamethyldisiloxane (*δ* = 6.53 ppm). ^31^P{^1^H} NMR spectra were externally referenced to chlorodiphenylphosphine (δ = +81.92 ppm). The solid-state NMR spectra were measured with a Bruker Avance 400 MHz NMR spectrometer. All solid-state NMR measurements were recorded with the Bruker HPDEC standard pulse program with high-power ^1^H decoupling (^31^P) and cross-polarization (^29^Si). Typically, for ^31^P{^1^H} MAS measurements, 1024 scans with a pulse delay of 7 s were recorded to result in spectra with decent S/N ratios within 2 h. For ^29^Si{^1^H} NMR measurements 7000 scans with a pulse delay of 6 s were recorded. ^31^P{^1^H} MAS spectra were externally referenced to ammoniumdihydrogen phosphate (δ = 0.81 ppm). ^29^Si CP/MAS spectra were externally referenced to tetrakis(trimethylsilyl)silane (δ = 8.62 ppm). The spectra were processed using exponential multiplication with a linebroadening factor of 80 Hz prior to FT. The ^31^P{^1^H} MAS halfwidth of **3i** was determined with no linebroadening. The halfwidth of **8i** ^31^P{^1^H} MAS was obtained with the minimal effective linebroadening of 30 Hz, and likewise **3i** ^29^Si CP/MAS with a linebroadening factor of 80 Hz.

### 4.3. X-Ray Diffraction

All details are provided in reference [19] and the Supplementary Materials.

### 4.4. Syntheses

**MeSi(CH_2_CH_2_PPh_2_)_3_ (1).** A flask was charged with trivinylmethylsilane (0.1692 g, 1.362 mmol), diphenylphosphine (2.945 g, 15.82 mmol), and AIBN (0.0552 g, 0.336 mmol, 1.92 mol%) and the reaction mixture was stirred for 72 h at 70 °C. Excess diphenylphosphine was vacuum-distilled from the product at 230 °C, yielding a viscous yellow oil. High purity was obtained by recrystallizing **1** from a 28 wt% solution in a 5:2 mixture of toluene and methanol by slow cooling. A colorless solid was obtained (0.7814 g, 1.144 mmol, 83.99% yield) with a mp of 98–101 °C, in accordance with the literature [13].

**^1^H NMR** (CDCl_3_, 500.13 MHz): *δ* (ppm) = 7.31–7.36 (m, 12H, H_aryl_), 7.28–7.31 (m, 18H, H_aryl_), 1.80–1.85 (m, 6H, PC*H*_2_), 0.55–0.61 (m, 6H, SiC*H*_2_), −0.02 (s, 3H, C*H*_3_); **^13^C{^1^H} NMR** (CDCl_3_, 125.77 MHz): *δ* (ppm) = 136.65 (d, ^1^*J*(^31^P-^13^C) = 14.2 Hz, C*_i_*), 132.69 (d, ^2^*J*(^31^P-^13^C) = 17.9 Hz, C*_o_*), 128.36 (s, C*_p_*), 128.36 (d, ^3^*J*(^31^P-^13^C) = 6.4 Hz, C*_m_*), 21.45 (d, ^1^*J*(^31^P-^13^C) = 14.2 Hz, P*C*H_2_), 10.85 (d, ^2^*J*(^31^P-^13^C) = 10.8 Hz, Si*C*H_2_), −5.69 (s, Si*C*H_3_); **^31^P{^1^H} NMR** (CDCl_3_, 202.28 MHz): *δ* (ppm) = −9.88 (s); **^29^Si{^1^H} NMR** (CDCl_3_, 79.37 MHz): *δ* (ppm) = 6.27 (q, ^3^*J*(^31^P-^29^Si) = 21.2 Hz) [28].

**MeOSi(CH_2_CH_2_PPh_2_)_3_ (2).** Trivinylmethoxysilane (0.1589 g, 1.133 mmol), diphenylphosphine (3.947 g, 21.20 mmol), AIBN (0.0801 g, 0.488 mmol, 2.14 mol%), and toluene (1.9 mL) were combined in a flask and stirred for 48 h at 80 °C. The excess of diphenylphosphine was vacuum-distilled from the reaction mixture at 230 °C and a viscous yellow oil was obtained. Three drops of diethyl ether were added and the mixture was cooled to 5 °C. After 18 h a colorless solid formed (0.6678 g, 0.9556 mmol, 84.34%). mp 75–77 °C.

**^1^H NMR** (CDCl_3_, 499.69 MHz): *δ* (ppm) = 7.33–7.38 (m, 12H, H*_o_*), 7.28–7.31 (m, 18H, H_aryl_), 3.31 (s, 3H, C*H*_3_), 1.85–1.90 (m, 6H, PC*H*_2_), 1.64–1.71 (m, 6H, SiC*H*_2_); **^13^C{^1^H} NMR** (CDCl_3_, 125.65 MHz): *δ* (ppm) = 138.69 (d, ^1^*J*(^31^P-^13^C) = 14.0 Hz, C*_i_*), 132.88 (d, ^2^*J*(^31^P-^13^C) = 18.0 Hz, C*_o_*), 128.73 (s, C*_p_*), 128.54 (d, ^3^*J*(^31^P-^13^C) = 6.6 Hz, C*_m_*), 50.94 (s, *C*H_3_), 20.75 (d, ^1^*J*(^31^P-^13^C) = 14.6 Hz, P*C*H_2_), 8.11 (d, ^2^*J*(^31^P-^13^C) = 11.3 Hz Si*C*H_2_); **^31^P{^1^H} NMR** (CDCl_3_, 202.28 MHz): *δ* (ppm) = −9.83 (s). **^29^Si{^1^H} NMR** (toluene-*d*_8_, 99.30 MHz): *δ* (ppm) = 15.35 (q, ^3^*J*(^31^P-^29^Si) = 22.1 Hz).

**EtOSi(CH_2_CH_2_PPh_2_)_3_ (3).** Trivinylethoxysilane (0.2774 g, 1.798 mmol), diphenylphosphine (3.966 g, 21.30 mmol), AIBN (0.0672 g, 0.409 mmol, 1.75 mol%), and toluene (1.3 mL) were placed in a flask and stirred together for 24 h at 70 °C. The mixture was cooled to RT and additional AIBN (0.547 g, 0.333 mmol, 1.40 mol%) was added. The reaction mixture was stirred for 48 h at 70 °C. The excess of diphenylphosphine was vacuum-distilled from the reaction mixture at 230 °C and a viscous yellow oil was obtained. After cooling to RT a colorless solid formed in quantitative yield (1.282 g, 1.798 mmol). mp 89–95 °C.

**^1^H NMR** (CDCl_3_, 400.09 MHz): *δ* (ppm) = 7.32–7.37 (m, 12H, H*_o_*), 7.27–7.31 (m, 18H, H_aryl_), 3.52 (q, ^3^*J*(^1^H-^1^H) = 7.0 Hz, 2H, OC*H*_2_), 1.84–1.89 (m, 6H, PC*H*_2_), 1.09 (t, ^3^*J*(^1^H-^1^H) = 7.0 Hz, 3H, C*H*_3_) 0.62–0.71 (m, 6H, SiC*H*_2_); **^13^C{^1^H} NMR** (CDCl_3_, 100.63 MHz): *δ* (ppm) = 138.76 (d, ^1^*J*(^31^P-^13^C) = 14.2 Hz, C*_i_*), 132.89 (d, ^2^*J*(^31^P-^13^C) = 18.0 Hz, C*_o_*), 128.71 (s, C*_p_*), 128.53 (d, ^3^*J*(^31^P-^13^C) = 6.5 Hz, C*_m_*), 58.82 (s, O*C*H_2_), 20.83 (d, ^1^*J*(^31^P-^13^C) = 14.6 Hz, P*C*H_2_), 18.69 (s, *C*H_3_), 8.50 (d, ^2^*J*(^31^P-^13^C) = 11.1 Hz, Si*C*H_2_); **^31^P{^1^H} NMR** (CDCl_3_, 161.96 MHz): *δ* (ppm) = −9.39 (s); **^29^Si{^1^H} NMR** (toluene-*d*_8_, 99.30 MHz): *δ* (ppm) = 13.31 (q, ^3^*J*(^31^P-^29^Si) = 22.2 Hz).

**[MeSi(CH_2_CH_2_PPh_2_)_3_]_2_(PdCl_2_)_3_ (4).** A solution of (PhCN)_2_PdCl_2_ (0.2771 g, 0.7224 mmol) in 60 mL of toluene was added dropwise to a toluene solution of **1** (0.5150 g, 0.7224 mmol). The mixture was stirred overnight at RT and yellow precipitate of **4** formed. The toluene was decanted, and the complex dissolved 230 mL of hot benzene and filtered while hot. The solvent was removed in *vacuo* and a yellow solid was obtained (0.6060 g, 0.3095 mmol, 64.27%). For growing crystals of sufficient quality for single crystal X-ray diffraction diethyl ether was layered over a DCM solution of **4**.

**^1^H NMR** (CDCl_3_, 499.69 MHz): *δ* (ppm) = 7.71–7.77 (m, 24H, H*_o_*), 7.42 (t, ^3^*J*(^1^H-^1^H) = 7.3 Hz, 12H, H*_p_*), 7.35 (t, ^3^*J*(^1^H-^1^H) = 7.3 Hz, 24H, H*_m_*), 2.37–2.44 (m, 12H, PC*H*_2_), 1.01–1.08 (m, 12H, SiC*H*_2_), −0.28 (s, 6H, CH_3_); **^13^C{^1^H} NMR** (CDCl_3_, 125.65 MHz): *δ* (ppm) = 133.95 (virtual t, ^1^*J*(^31^P-^13^C) = 5.9 Hz, C*_i_*), 130.47 (virtual t, ^2^*J*(^31^P-^13^C) = 22.1 Hz, C*_o_*), 130.18 (s, C*_p_*), 128.17 (virtual t, ^3^*J*(^31^P-^13^C) = 4.8 Hz, C*_m_*), 19.82 (virtual t, ^1^*J*(^31^P-^13^C) = 12.3 Hz, P*C*H_2_), 7.29 (s, Si*C*H_2_), −6.32 (s, Si*C*H_3_); **^31^P{^1^H} NMR** (CDCl_3_, 202.34 MHz): *δ* (ppm) = 22.34 (s). mp 220 °C.

**[MeOSi(CH_2_CH_2_PPh_2_)_3_]_2_(PdCl_2_)_3_ (5).** A solution of **2** (82.1 mg, 0.117 mmol) in 25 mL of a 5:1 mixture of DCM with MeCN was combined with (PhCN)_2_PdCl_2_ (61.0 mg, 0.159 mmol) and stirred for 2 h at RT. The solution turned yellow. It was filtered and the solvent was removed in *vacuo*. The residue was washed with 10 mL toluene, yielding **5** as a yellow solid (172.5 mg, 0.08939 mmol, 76.4%). For growing crystals of sufficient quality for single-crystal X-ray diffraction diethyl ether was layered over a DCM solution of **5**.

**^1^H NMR** (CDCl_3_, 499.69 MHz): *δ* (ppm) = 7.70–7.76 (m, 24H, H*_o_*), 7.42 (t, ^3^*J*(^1^H-^1^H) = 7.2 Hz, 12H, H*_p_*), 7.36 (t, ^3^*J*(^1^H-^1^H) = 7.3 Hz, 24H, H*_m_*), 3.05 (s, 6H, OC*H*_3_) 2.37–2.47 (m, 12H, PC*H*_2_), 1.08–1.16 (m, 12H, SiC*H*_2_); **^31^P{^1^H} NMR** (CDCl_3_, 162.00 MHz): *δ* (ppm) = 21.71 (s).

**[EtOSi(CH_2_CH_2_PPh_2_)_3_]_2_(PdCl_2_)_3_ (6).** (MeCN)_2_PdCl_2_ (22.9 mg, 0.0883 mmol) was added to a solution of **3** (42.0 mg, 0.0589 mmol) in 10 mL DCM and the reaction mixture was stirred overnight at RT. The yellow solution was filtered and the solvent was removed in *vacuo*. The solid yellow residue was washed with 15 mL diethyl ether and pure **6** was obtained (83.1 mg, 0.0425 mmol, 72.1%).

**^1^H NMR** (CDCl_3_, 499.69 MHz): δ (ppm) = 7.72–7.77 (m, 24H, H*_o_*), 7.42 (t, ^3^*J*(^1^H-^1^H) = 7.3 Hz, 12H, H*_p_*), 7.36 (t, ^3^*J*(^1^H-^1^H) = 7.4 Hz, 24H, H*_m_*), 3.24 (q, ^3^*J*(^1^H-^1^H) = 7.0 Hz, 4H, OC*H*_2_) 2.44–2.49 (m, 12H, PC*H*_2_), 1.04–1.11 (m, 12H, SiC*H*_2_), 0.83 (t, ^3^*J*(^1^H-^1^H) = 7.0 Hz. 6H, C*H*_3_); **^31^P{^1^H} NMR** (CDCl_3_, 202.34 MHz): *δ* (ppm) = 21.68 (s).

**Representative procedure for the immobilization of 3 (3i).** A flask was charged with **3** (0.2979 g, 0.4179 mmol), toluene (30 mL) and dried silica (3.1137 g) and the mixture was stirred for 58 h at 70 °C. The supernatant was decanted and the silica was washed three times with 20 mL aliquots of toluene, then three times with 20 mL of acetone. The supernatant and washings were stripped of the solvent *in vacuo*, leaving 0.1367 g of residue (51.7 mg, 0.0725 mmol **3** per g silica), corresponding to about 5.8 molecules per 100 nm^2^ of surface area).

**^31^P{^1^H} MAS NMR** (162.00 MHz, 10 kHz rotational frequency): *δ* (ppm) = −9.74 (∆ν_1/2_ = 210 Hz). **^29^Si CP/MAS NMR** (79.51 MHz, 4 kHz rotational frequency): *δ* (ppm) = 9.45 (∆ν_1/2_ = 330 Hz).

**Representative procedure for generating the immobilized Pd complex using 3i with maximal surface coverage.** A flask was charged with **3i** (1.1306 g, 51.7 mg **3** per g silica, 0.0820 mmol **3i**), (MeCN)_2_PdCl_2_ (32.8 mg, 0.126 mmol, 1.54 eq), and acetonitrile (30 mL) and was stirred for 18 h at RT. The supernatant was decanted and the silica was washed three times with 20 mL aliquots of acetonitrile.

**^31^P{^1^H} MAS NMR** (162.00 MHz, 10 kHz rotational frequency): *δ* (ppm) = 31.3 (∆ν_1/2_ = 1.10 kHz).

**Representative procedure for generating the immobilized Cu complex using 3i.** A flask was charged with **3i** with 23% surface coverage (0.1105 g, 11.8 mg **3** per g silica, 0.00183 mmol **3i**), CuCl (2.5 mg, 0.025 mmol, 14 eq), and acetonitrile (5 mL) and the reaction mixture was stirred for 18 h at RT. The supernatant was decanted and the silica was washed three times with 5 mL aliquots of acetonitrile.

**^31^P{^1^H} HRMAS NMR** (acetonitrile, 162.00 MHz, 2 kHz rotational frequency): *δ* (ppm) = −3.5 (∆ν_1/2_ = 510 Hz).

## Figures and Tables

**Table 1:** Selected bond angles in trinuclear metal complexes (°). **pOp_3_** stands for the ligand O=P(CH_2_CH_2_PPh_2_)_3_, **pOp_3_S_3_** for O=P(CH_2_CH_2_P(S)Ph_2_)_3_ and **etp** for the ligand CH_3_C(CH_2_CH_2_PPh_2_)_3_ [21,22]. **L** = P, except for **pOp_3_S_3_** where **L** = S.

Complex	L–M–L (°)		
**4**	165.68 (3)	175.79 (3)	176.09 (3)
**5a**	174.22 (3)	174.71 (3)	178.24 (3)
**5b**	165.45 (3)	176.33 (3)	176.45 (3)
**7**	177.22 (4)	179.02 (4)	179.31 (5)
**Pd_3_I_6_(pOp_3_)_2_** [21]	175.8 (2)	176.4 (2)	178.2 (2)
**Pd_3_Cl_6_(pOp_3_S_3_)_2_** [21]	168.1 (1)	168.1 (1)	168.1 (1)
**Ni_3_Cl_6_(etp)_2_** [22]	167.62	171.80	176.54

**Table 2:** Selected bond angles in trinuclear metal complexes (°). **pOp_3_** stands for the ligand O=P(CH_2_CH_2_PPh_2_)_3_, **pOp_3_S_3_** for O=P(CH_2_CH_2_P(S)Ph_2_)_3_ and **etp** for CH_3_C(CH_2_CH_2_PPh_2_)_3_. **X** = Cl, I [21,22].

Complex	X–M–X		
**4**	178.76 (3)	173.04 (4)	171.76 (4)
**5a**	168.83 (5)	176.18 (4)	176.89 (4)
**5b**	179.23 (3)	174.14 (3)	172.24 (3)
**7**	174.09 (5)	176.63 (5)	174.20 (5)
**Pd_3_I_6_(pOp_3_)_2_** [21]	160.26 (7)	172.6 (2)/158.5 (2)	169.38 (7)
**Pd_3_Cl_6_(pOp_3_S_3_)_2_** [21]	179.5 (1)	179.5 (1)	179.5 (1)
**Ni_3_Cl_6_(etp)_2_** [22]	161.97	166.85	166.31

**Table 3:** Selected dihedral angles in trinuclear metal complexes (°). **pOp_3_** stands for the ligand O=P(CH_2_CH_2_PPh_2_)_3_, **pOp_3_S_3_** for O=P(CH_2_CH_2_P(S)Ph_2_)_3_ and **etp** for CH_3_C(CH_2_CH_2_PPh_2_)_3_ [21,22]. **L** = P, except for **pOp_3_S_3_** where **L** = S. **Center** = Si (**4**, **5a**, **5b**, **7**), P for the other Pd complexes, and C for the Ni complex.

Complex	Dihedral Angles L–Center–Center–L (°)		
**4**	2.11 (2)	2.93 (3)	8.14 (3)
**5a**	4.41 (3)	8.95 (3)	12.68 (3)
**5b**	2.91 (3)	1.90 (2)	7.40 (3)
**7**	5.43 (3)	6.27 (3)	11.72 (3)
**Pd_3_I_6_(pOp_3_)_2_** [21]	2.0 (1)	0.6 (1)	0.8 (1)
**Pd_3_Cl_6_(pOp_3_S_3_)_2_** [21]	50.68	50.68	50.68
**Ni_3_Cl_6_(etp)_2_** [22]	0.37	0.10	2.35

**Table 4:** Selected center-center and metal-metal distances in the listed trinuclear complexes (Å). **pOp_3_** stands for the ligand O=P(CH_2_CH_2_PPh_2_)_3_, **pOp_3_S_3_** for O=P(CH_2_CH_2_P(S)Ph_2_)_3_ and **etp** for CH_3_C(CH_2_CH_2_PPh_2_)_3_ [21,22]. **M** = Pd, Ni. **Center** = Si (**4**, **5a**, **5b**, **7**), P for the other Pd complexes, and C for the Ni complex.

Complex	Center–Center	M–M		
**4**	7.364 (1)	7.4963 (8)	6.8894 (8)	7.5769 (7)
**5a**	8.363 (1)	7.0903 (6)	7.2621 (7)	7.1109 (6)
**5b**	7.453 (1)	7.4188 (5)	7.8960 (6)	7.4932 (5)
**7**	8.530 (2)	7.1131 (8)	6.8095 (7)	7.1727 (7)
**Pd_3_I_6_(pOp_3_)_2_** [21]	6.882 (9)	7.73 (1)	7.35 (1)	7.325 (8)
**Pd_3_Cl_6_(pOp_3_S_3_)_2_** [21]	4.85 (9)	7.616 (8)	7.62 (1)	7.616 (7)
**Ni_3_Cl_6_(etp)_2_** [22]	6.051	7.783	6.893	7.27

## Data Availability

CCDC 2201608 (**4**), 2201218 (**5a**), 2422836 (**5b**), and 2201217 (**7**) contain the crystallographic data for this paper. These data can be obtained free of charge via www.ccdc.cam.ac.uk/data_request/cif, or by emailing data_request@ccdc.cam.ac.uk, or by contacting The Cambridge Crystallographic Data Centre, 12 Union Road, Cambridge CB2 1EZ, UK. Available online: www.ccdc.cam.ac.uk/data_request/cif (accessed on 1 January 2025).

## Supplementary Materials

The following supporting information can be downloaded at: https://www.mdpi.com/article/10.3390/molecules30071616/s1, Figure S1: ^31^P{^1^H} NMR spectra of **1**-**6** in the indicated solvents; Figure S2: ^1^H NMR spectrum of **1** in C_6_D_6_. The asterisks denote traces of HPPh_2_ and s stems from a solvent impurity; Figure S3: ^1^H NMR spectrum of **2** in C_6_D_6_; Figure S4: ^1^H NMR spectrum of **2** in CDCl_3_; Figure S5: ^1^H NMR spectrum of **3** in CDCl_3_; Figure S6: ^13^C{^1^H} NMR spectrum of **2** in CDCl_3_; Figure S7: ^13^C{^1^H} NMR spectrum of **3** in CDCl_3_; Figure S8: ^13^C{^1^H} NMR spectrum of **4** in CDCl_3_; Figure S9: Single crystal X-ray structure of one molecule of **5b**. View from the side (left) and along the Si···Si axis (right). Hydrogen atoms and disordered solvent molecules are omitted for clarity; Figure S10: Unit cell of **5b**. Hydrogen atoms and disordered solvent molecules are omitted for clarity; Figure S11: ^31^P{^1^H} MAS NMR spectrum of polycrystalline **4** recorded at 4 kHz spinning frequency. Rotational sidebands are indicated with asterisks; Figure S12: Unit cell of **7**. Hydrogen atoms and disordered solvent molecules are omitted for clarity; Table S1: Crystallographic data for **4**, **5a**, **5b**, and **7**; Figure S13: ^31^P{^1^H} MAS NMR spectra of **3i** (top) and of **3i** after oxidation with hydrogen peroxide (bottom) at the indicated rotational speeds. References [29,30,31,32,33,34,35,36] are cited in the supplementary materials.
