# Supplementary material for: Molecular and Immobilized Tripodal Phosphine Ligands and Their Trinuclear Palladium Complexes
Source: Molecules. 2025 Apr 4;30(7):1616. doi: 10.3390/molecules30071616 (PMC11990150; doi:10.3390/molecules30071616)
Supplement: Supplementary file 1 [file molecules-30-01616-s001.zip › molecules-3527412-supplementary.pdf]

Electronic Supplementary Material (ESI)

Molecular and Immobilized Tripodal Phosphine Ligands and their Trinuclear Palladium Complexes

Maxwell R. Kimball, Kyle J. Cluff, Nattamai Bhuvanesh, Janet Blümel*

Submitted Feb. 2025

Department of Chemistry, Texas A&M University, College Station, TX, 77842-3012, USA.
Email: bluemel@tamu.edu

I. Solution NMR Spectroscopy

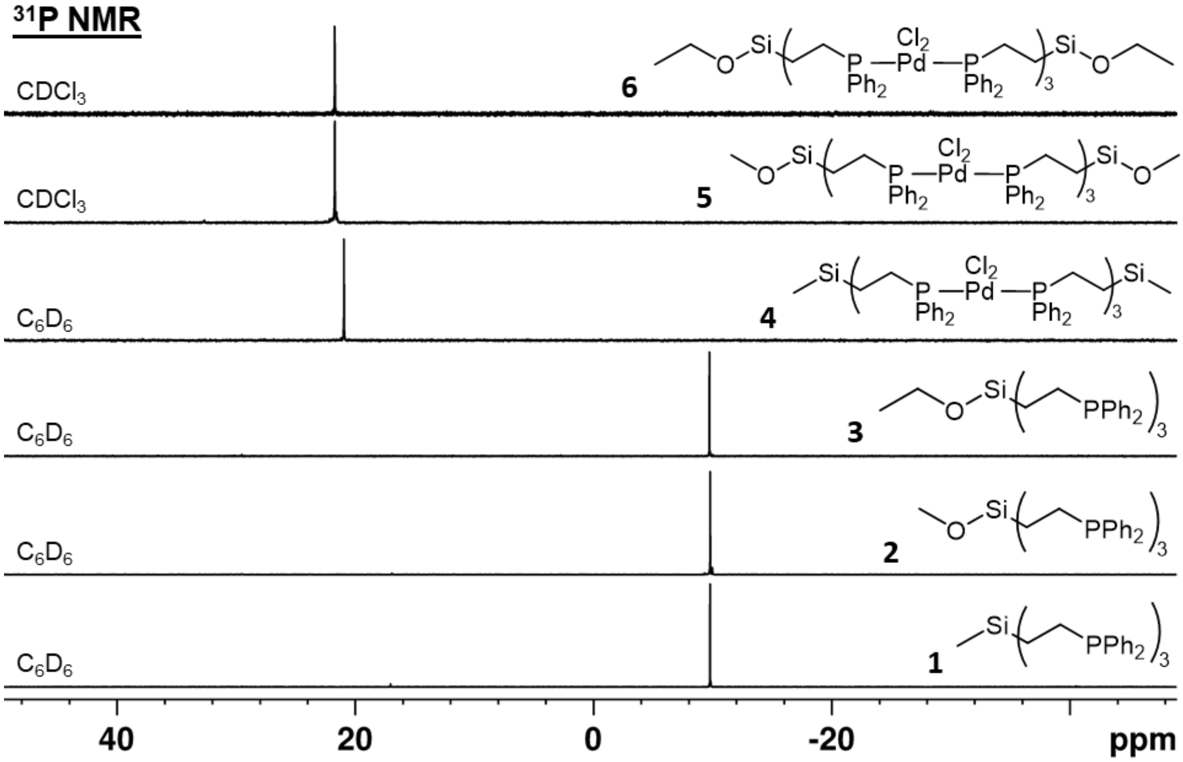

Figure S1. ^{31}P{^{1}H} NMR spectra of **1-6** in the indicated solvents.

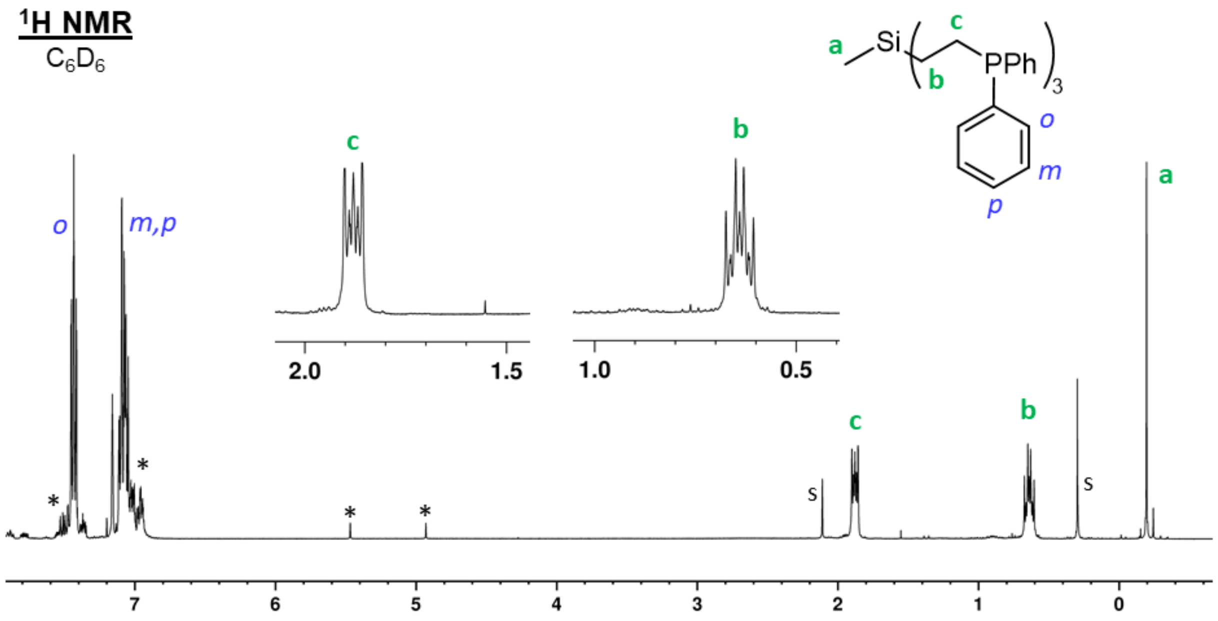

Figure S2. ^{1}H NMR spectrum of **1** in C_6D_6. The asterisks denote traces of $HPPh_2$ and s stems from a solvent impurity.

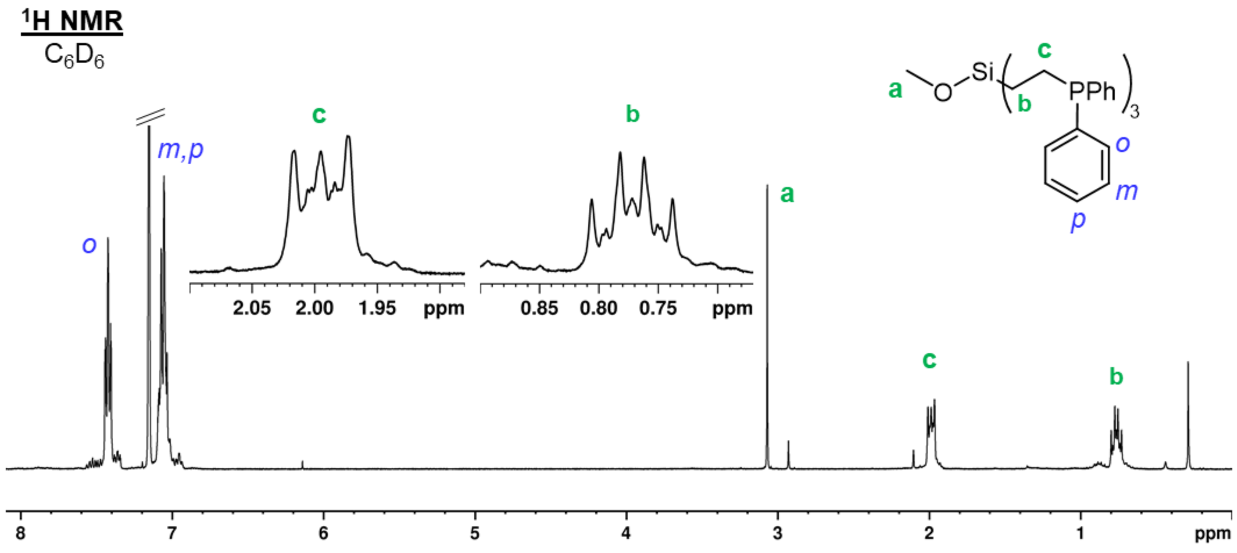

Figure S3. ^{1}H NMR spectrum of **2** in C_6D_6.

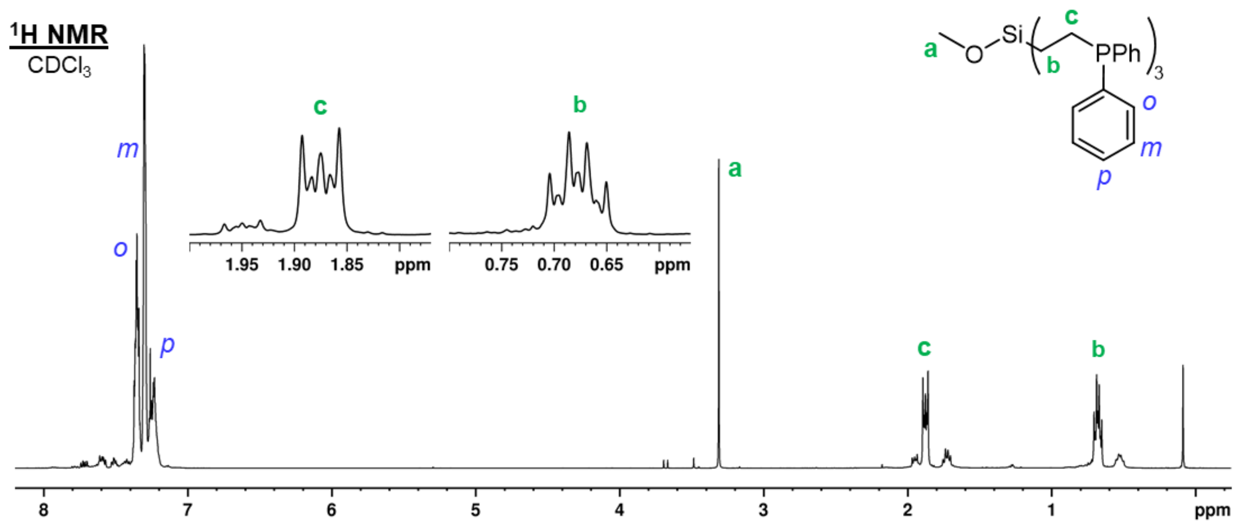

Figure S4. ^{1}H NMR spectrum of **2** in $CDCl_3$.

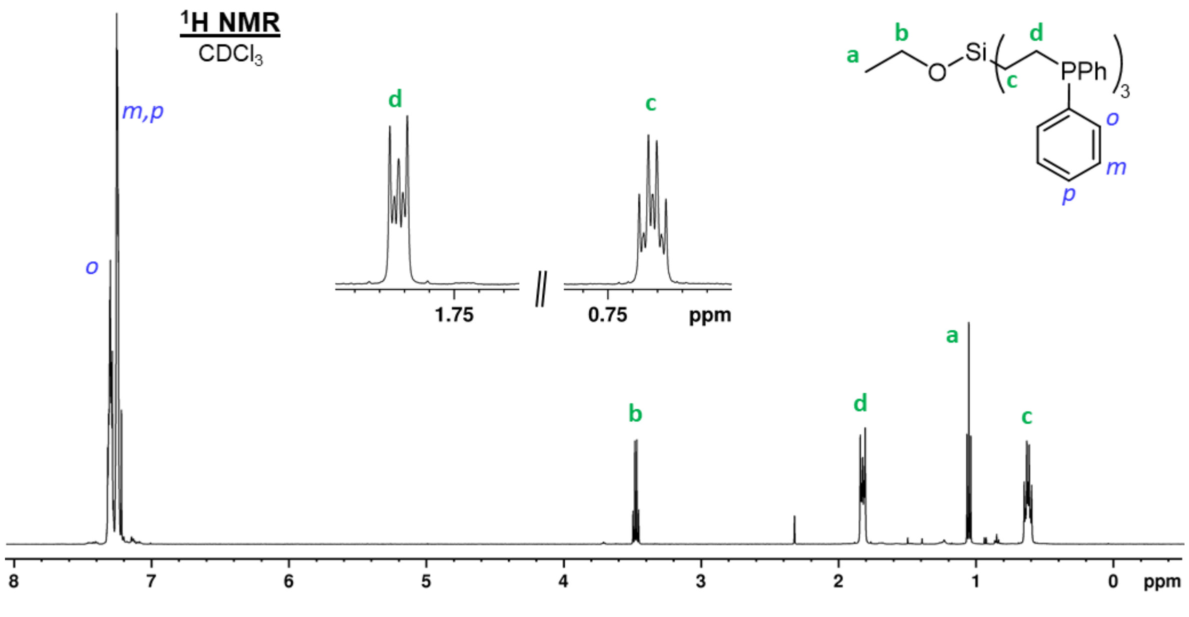

Figure S5. ^{1}H NMR spectrum of **3** in $CDCl_3$.

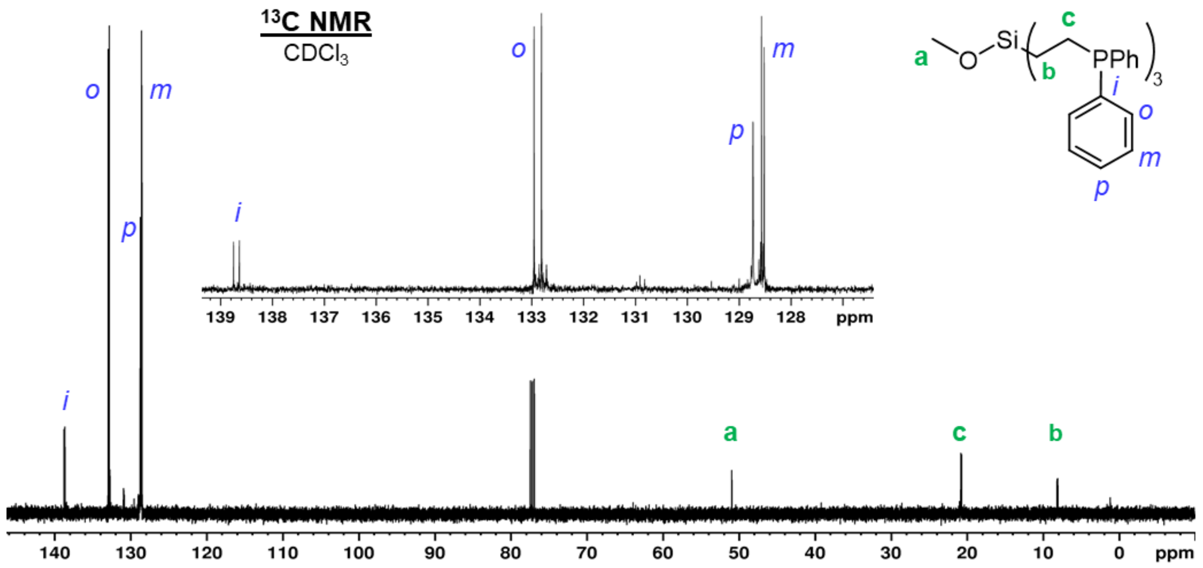

Figure S6. $^{13}C\{^1H\}$ NMR spectrum of **2** in $CDCl_3$.

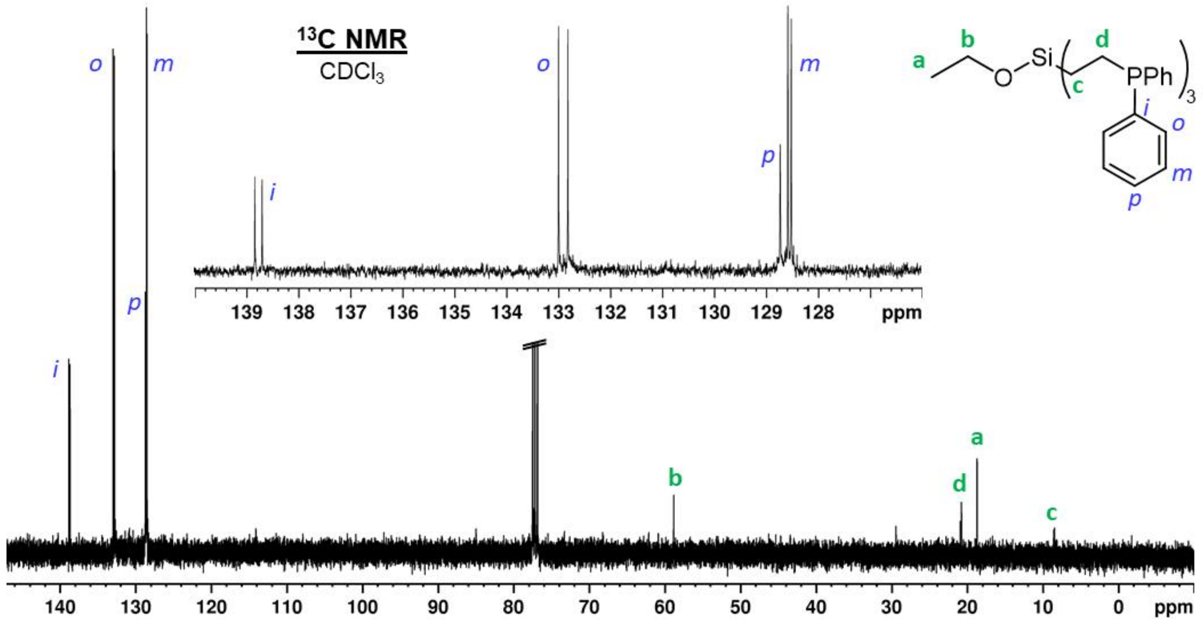

Figure S7. $^{13}C\{^1H\}$ NMR spectrum of **3** in $CDCl_3$.

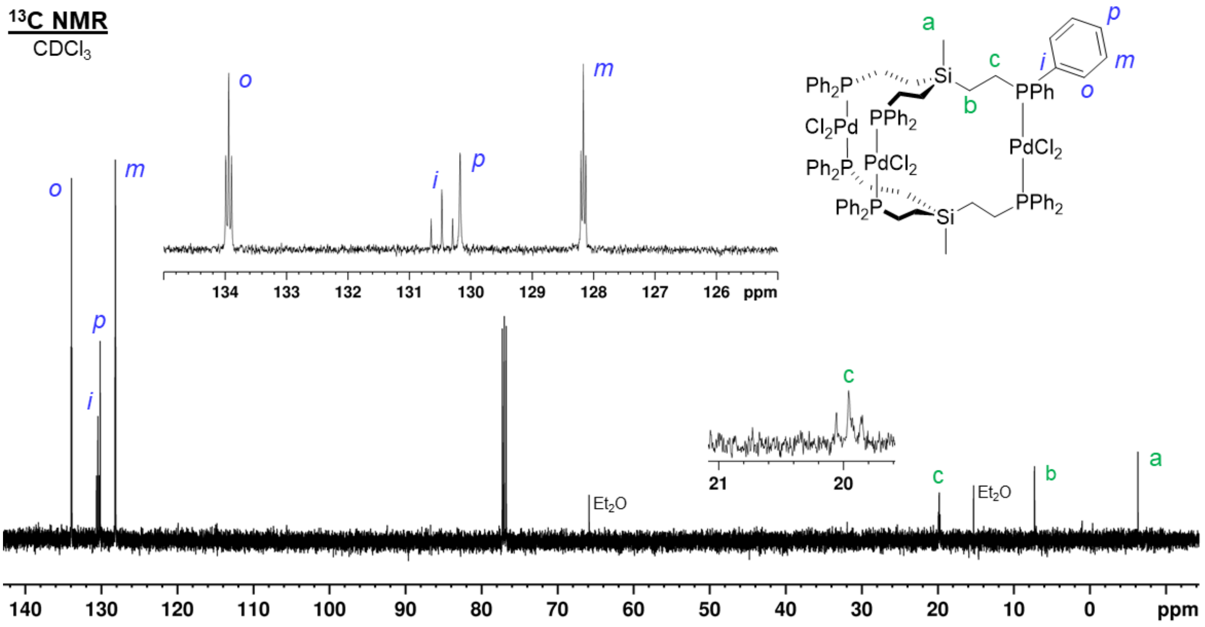

Figure S8. $^{13}C\{^1H\}$ NMR spectrum of **4** in $CDCl_3$.

II. X-Ray Crystallography and Solid-State NMR Spectroscopy

Compound 4. A solution of **4** in dichloromethane was layered with an equal volume of diethyl ether. A yellow block with very well-defined faces from a representative sample of crystals of the same habit was collected and data were obtained as outlined in Table **S1**. The X-ray radiation employed was generated from a Cu-Iμs X-ray tube (K_α = 1.5418 Å with a potential of 50 kV and a current of 1.0 mA). 45 data frames were taken at widths of 1.0°. These reflections were used to determine the unit cell. The unit cell was verified by examination of the *h k l* overlays on several frames of data. No super-cell or erroneous reflections were observed. After careful examination of the unit cell, an extended data collection procedure (39 sets) was initiated using omega and phi scans.

Integrated intensity information for each reflection was obtained by reduction of the data frames with the program *APEX3*.[S1] The integration method employed a three-dimensional profiling algorithm and all data were corrected for Lorentz and polarization factors, as well as for crystal decay effects. Finally, the data was merged and scaled to produce a suitable data set. The absorption correction program *SADABS*[S2] was employed to correct the data for absorption effects.

Systematic reflection conditions and statistical tests of the data were used to determine the space group. A solution was obtained readily using *XT/XS* in *APEX3*.[S1,S3,S4,S5] Approximately two molecules of DCM were found solvated in three different partially occupied positions in the asymmetric unit. Also, difference Fourier maps indicated the presence of disordered, solvated water and diethyl ether, which could not be successfully modeled. For the final least squares refinement,

these solvent molecules were MASKed using OLEX2, which agreed closely to a molecule each of ether and water in the asymmetric unit. Hydrogen atoms were placed in idealized positions and were set riding on the respective parent atoms.[S6] All non-hydrogen atoms were refined with anisotropic thermal parameters. Thermal ellipsoids on some of the peripheral phenyl groups ([C51-C56]; [C61-C66, C67-C72]) show significant elongation suggesting disorder. They were modeled between two positions each with an occupancy ratio of 0.49:0.51 and 0.76:0.24, respectively. Appropriate restraints and constraints were added to keep the bond distances, angles, and thermal ellipsoids of the disordered groups meaningful.

The structure was refined (weighted least squares refinement on F^2) to convergence.[S3,S4,S5,S7] *Olex2* and *Mercury* were employed for the final data presentation and structure plots.[S7,S8]

Compound 5. THF solvate 5a: A solution of **5** in a 1:5 mixture of dichloromethane and tetrahydrofuran was combined with 4 parts diethyl ether and left to stand at RT for 3 days. A yellow block with very well-defined faces from a representative sample of crystals of the same habit was collected, data were obtained as outlined in Table **S1**, and the structure was solved as described for **4**. The X-ray radiation employed was generated from a Cu-Iµs X-ray tube (Kα = 1.5418 Å with a potential of 50 kV and a current of 1.0 mA). 45 data frames were taken at widths of 1.0°. These reflections were used to determine the unit cell. The unit cell was verified by examination of the *h k l* overlays on several frames of data. No super-cell or erroneous reflections were observed. After careful examination of the unit cell, an extended data collection procedure (41 sets) was initiated using omega and phi scans.

Hydrogen atoms were placed in idealized positions and were set riding on the respective parent atoms. All non-hydrogen atoms were refined with anisotropic thermal parameters.

Half a molecule of THF was clearly located next to Si(1)-O(1)-C(1)H3, and the disorder was modeled. However, solvent molecules (THF, Et_2O, and CH_2Cl_2) were present partially occupied and disordered, which could not be modeled. The latter were MASK'ed using Olex2. A solvent mask was calculated and 244 electrons were found in a volume of 1140 $Å^3$ in 2 voids per unit cell. This is consistent with the presence of 1.5[C_4H_8O] per Formula Unit which account for 240 electrons per unit cell. [Note: Although we have assumed the MASK'ed solvent is THF in the Moiety formula, there were indications that a mixture of THF, Et_2O, and CH_2Cl_2 were present, partially occupied and disordered]. Final formula: $C_{86}H_{90}Cl_6O_2P_6Pd_3Si_2\cdot0.5(C_4H_8O)\cdot1.5[C_4H_8O]$. The solvent in the square brackets represent the partially occupied and disordered solvents as described above.

Compound 5. Diethyl ether solvate 5b: A solution of **6** in dichloromethane was layered with diethyl ether containing a small amount of methanol. A yellow block with very well-defined faces from a representative sample of crystals of the same habit was collected, data were obtained as outlined in Table **S1**, and the structure was solved as described for **4**. The X-ray radiation employed was generated from a Cu-Iµs X-ray tube (K_α = 1.5418 Å with a potential of 50 kV and a current of 1.0 mA). 90 data frames were taken at widths of 1.0°. These reflections were used to determine the unit

cell. The unit cell was verified by examination of the *h k l* overlays on several frames of data. No super-cell or erroneous reflections were observed. After careful examination of the unit cell, an extended data collection procedure (46 sets) was initiated using omega and phi scans.

Hydrogen atoms were placed in idealized positions and were set riding on the respective parent atoms. All non-hydrogen atoms were refined with anisotropic thermal parameters.

Systematic reflection conditions and statistical tests of the data were used to determine the space group. A solution was obtained readily using *XT/XS* in *APEX3*.[S1,S3,S4,S5] Residual electron density peaks suggested four locations for solvent molecules, and indicated disorder in all four locations. The geometry of the peaks indicated that two of the sites have mixed ether/DCM disorder. Also, upon refinement of the solvent model, the thermal ellipsoids indicated that only two of the solvent sites were fully occupied. For the final least squares refinement cycles, the individual solvent occupancies were fixed (considering the meaningful chemical neighboring environments) to the closest refined values (rounded to hundredths) to avoid minor changes in the occupancies (and hence Alerts in the CHECKCIF and losing chemical meaningfulness). To confirm our solvent model further, we removed all the solvents and used MASK using OLEX2 which resulted in 148 e^- in the solvent mask. This agrees very well with our formula [147 e^- for solvents]: $C_{86}H_{90}Cl_6O_2P_6Pd_3Si_2 \cdot 2.23(CH_2Cl_2) \cdot 1.27(C_4H_{10}O)$. Our attempts to collect the data using a larger crystal on Bruker Quest (with Mo radiation) agreed well with the results reported here. However, the quality of the larger crystal was not good enough to model the solvent disorder [But, MASK yielded 152 e^- for solvents]. The structure was refined (weighted least squares refinement on F^2) to convergence.[S3,S4,S5,S7] Elongated thermal ellipsoids of three of the phenyl groups indicated disorder and were modeled between two positions each. Appropriate restraints and constraints were added for the disordered groups to keep the bond distances, angles, and thermal ellipsoids meaningful. Absence of additional symmetry or void were confirmed using PLATON (ADDSYM).[S6] The structure was refined (weighted least squares refinement on F^2) to convergence.[S3,S4,S5,S7]

Compound 7. A solution of **6** in wet dichloromethane was combined with diethyl ether by vapor diffusion. A yellow block with very well-defined faces from a representative sample of crystals of the same habit was collected, data were obtained as outlined in Table **S1**, and the structure was solved as described for **4**. The X-ray radiation employed was generated from a Cu- Iμs X-ray tube (K_α = 1.5418 Å with a potential of 50 kV and a current of 1.0 mA). 45 data frames were taken at widths of 1.0°. These reflections were used to determine the unit cell. The unit cell was verified by examination of the *h k l* overlays on several frames of data. No super-cell or erroneous reflections were observed. After careful examination of the unit cell, an extended data collection procedure (27 sets) was initiated using omega and phi scans.

Two molecules, ether and DCM, were found solvated. Hydrogen atoms were placed in idealized positions and were set riding on the respective parent atoms. All non-hydrogen atoms were refined with anisotropic thermal parameters. Elongated ellipsoids and nearby residual electron density peaks

suggested the solvated DCM was disordered and was modeled between two positions with an occupancy ratio 0.76:0.24.

Further elongation of the ellipsoids on DCM, some of the phenyl groups, and the residual electron densities near the ether indicated more disorder. Our efforts to model the disorder not only increased the number of refined parameters and restraints/constraints, but the improvement in the reliability factors were minimal. For the final refinement cycles the disorder of these groups was not modeled.

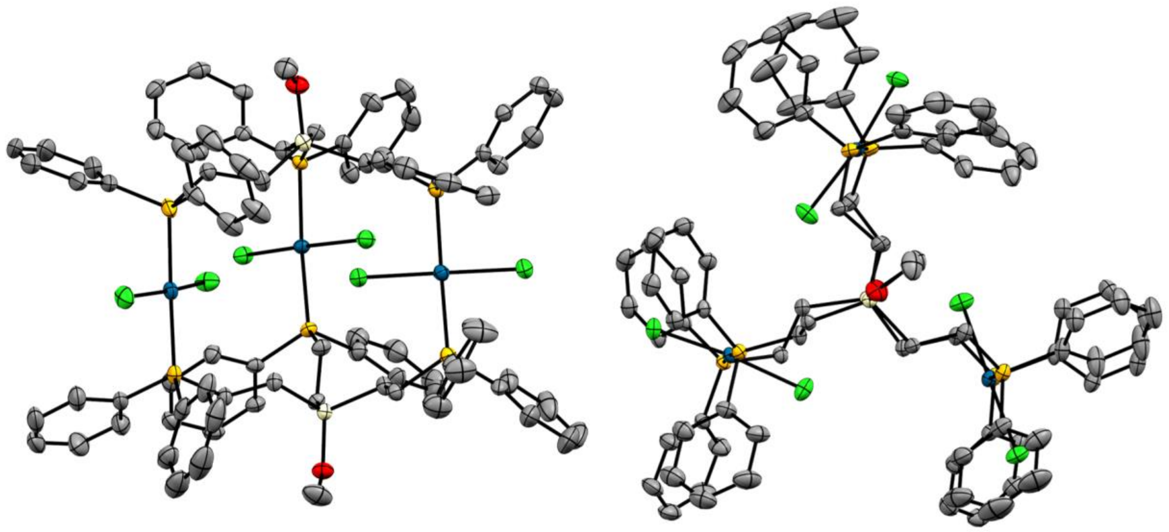

Figure S9. Single crystal X-ray structure of one molecule of **5b**. View from the side (left) and along the Si···Si axis (right). Hydrogen atoms and disordered solvent molecules are omitted for clarity.

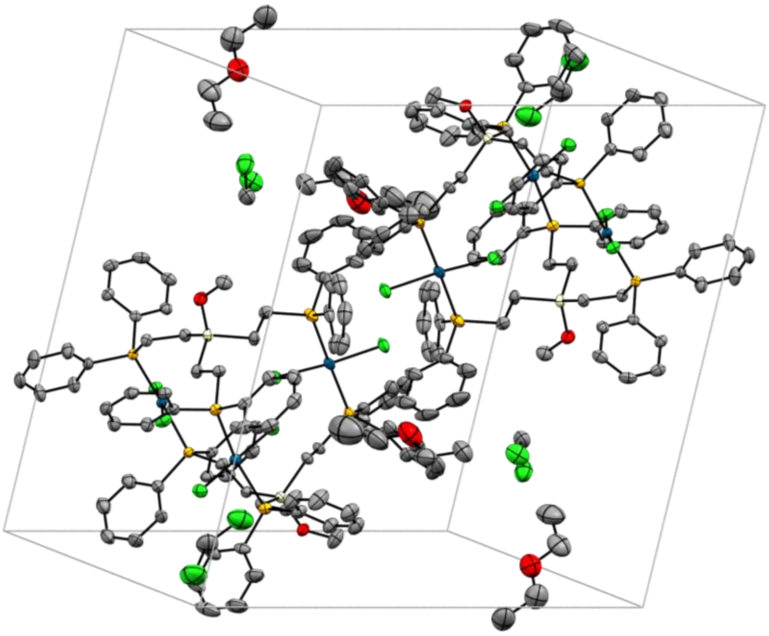

Figure S10. Unit cell of **5b**. Hydrogen atoms and disordered solvent molecules are omitted for clarity.

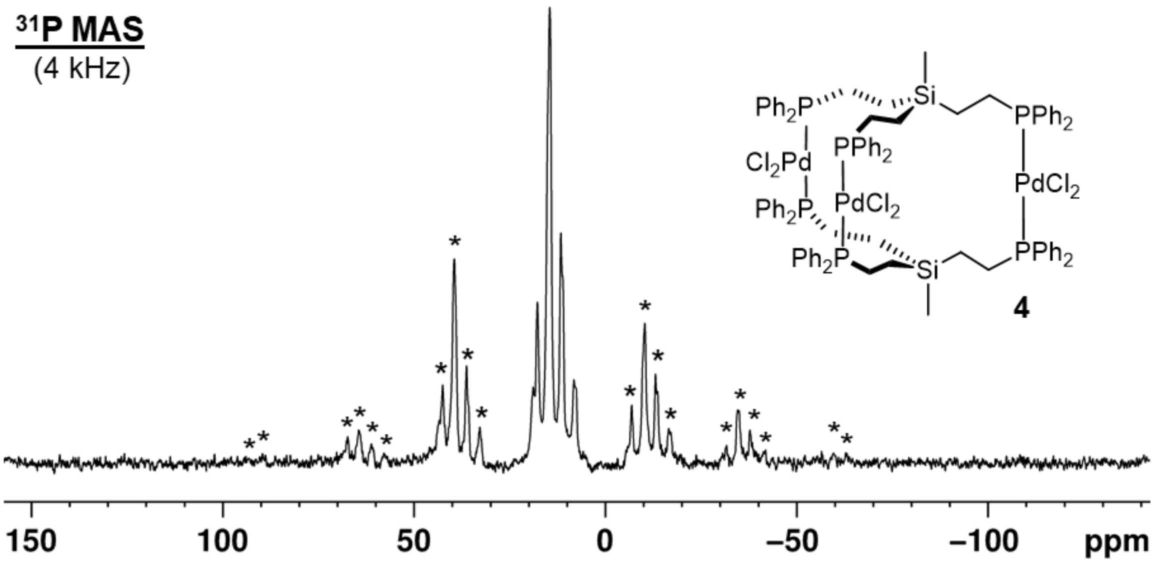

Figure S11. $^{31}P\{^{1}H\}$ MAS NMR spectrum of polycrystalline **4** recorded at 4 kHz spinning frequency. Rotational sidebands are indicated with asterisks.

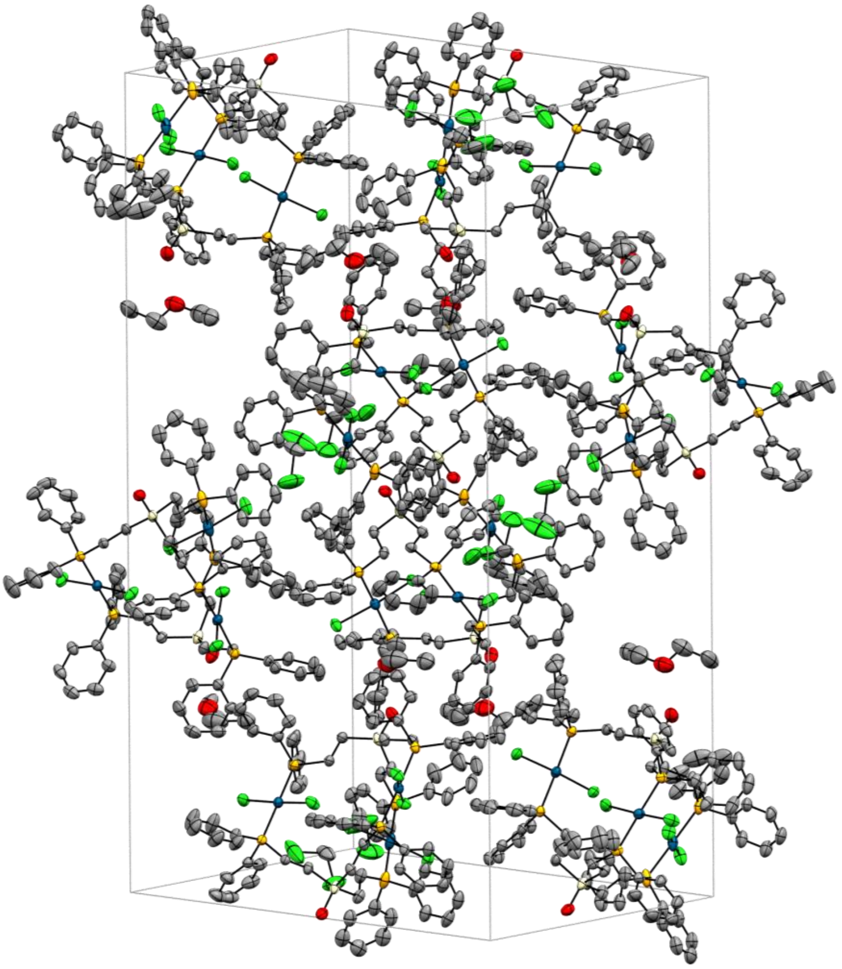

Figure S12. Unit cell of **7**. Hydrogen atoms and disordered solvent molecules are omitted for clarity.

Table S1. Crystallographic data for **4**, **5a**, **5b**, and **7**.

	**4**	**5a**	**5b**	**7**
Deposition #	CCDC 2201608	CCDC 2201218	CCDC 2422836	CCDC 2201217
empirical formula	$C_{87.97}H_{93.47}Cl_{9.95}P_6Pd_3Si_2$	$C_{88}H_{94}Cl_6O_{2.5}P_6Pd_3Si_2$	$C_{93.31}H_{107.16}Cl_{10.46}P_6Pd_3Si_2$	$C_{89}H_{94}Cl_8O_3P_6Pd_3Si_2$
formula weight	2064.90	1965.53	2212.99	2060.47
temperature [K]	110.0	110.0	110.0	110.0
diffractometer	Bruker Venture	Bruker Venture	Bruker Venture	Bruker Venture
wavelength [Å]	1.54178	1.54178	1.54178	1.54178
crystal system	Triclinic	Monoclinic	Triclinic	Orthorhombic
space group	*P-1*	$P2_1/c$	*P-1*	*P*bca
unit cell dimensions:				
a [Å]	16.7748(14)	22.7584(4)	16.8159(3)	24.4385(5)
b [Å]	17.5145(15)	21.1680(4)	17.5267(3)	17.6712(3)
c [Å]	20.5889(17)	20.5481(4)	20.4310(3)	42.8516(8)
α [°]	104.206(4)	90	103.6750(10)	90
β [°]	92.711(4)	107.1840(10)	93.7810(10	90
γ [°]	117.102(4)	90	117.3580(10)	90
V [Å^3]	5130.7(8)	9457.2(3)	5089.01(15)	18505.8(6)
Z	2	4	2	8
ρ_{calc} [Mg/m^3]	1.337	1.380	1.444	1.479
μ [mm^{-1}]	7.988	7.655	8.235	8.374
F(000)	2093	4000	2254	8384
crystal size [mm^3]	0.184 × 0.042 × 0.034	0.047 × 0.043 × 0.022	0.082 × 0.031 × 0.024	0.114 × 0.102 × 0.084
Θ limit [°]	3.144 to 70.356	2.032 to 70.209	2.272 to 70.157	2.743 to 70.175
index range (*h*, *k*, *l*)	−20, 20; −21, 21; −25, 25	−25, 27; −25, 25; −25, 25	−20, 20; −21, 21; −24, 24	−29, 29; −18, 21; −52, 52
reflections collected	152906	252049	171641	313123
independent reflections	19476	17970	19359	17584
R(int)	0.0451	0.0529	0.0529	0.0428]
completeness to Θ	99.9 %	100.0 %	100.0 %	100.0 %
max. and min. transmission	0.3841 and 0.1803	0.3841 and 0.2387	0.3841 and 0.2501	0.1891 and 0.0168
data / restraints / parameters	19476 / 875 / 1100	17970 / 69 / 1003	19359 / 1192 / 1312	17584 / 22 / 1026
goodness-of-fit on F^2	1.054	1.052	1.025	1.023
R indices (final) [$I > 2\sigma(I)$]				
R_1	0.0410	0.0452	0.0331	0.0493
wR_2	0.1182	0.1241	0.0917	0.01193
R indices (all data)				
R_1	0.0433	0.0489	0.385	0.0518
wR_2	0.1203	0.1295	0.0948	0.1215
largest diff. peak and hole [eÅ^{-3}]	2.522 and −1.109	1.807 and −1.320	0.997 and −1.277	1.862 and -1.248

References

[S1] Bruker. *APEX3*; Bruker AXS Inc.: Madison, Wisconsin, USA, 2015.

[S2] Bruker. *SADABS*; Bruker AXS Inc.: Madison, WI, USA, 2001.

[S3] Sheldrick, G.M. A short history of *SHELX*. *Acta Crystallogr. Sect. A* **2008**, *A64*, 112–122. https://doi.org/10.1107/s0108767307043930.

[S4] Sheldrick, G.M. *SHELXT*—Integrated space-group and crystal-structure determination. *Acta Crystallogr. Sect. A Found. Adv.* **2015**, *71*, 3–8. https://doi.org/10.1107/S2053273314026370.

[S5] Sheldrick, G.M. Crystal structure refinement with *SHELXL*. *Acta Crystallogr. Sect. C Struct. Chem.* **2015**, *71*, 3–8. https://doi.org/10.1107/s2053229614024218.

[S6] Spek, A.L. Structure validation in chemical crystallography. *Acta Crystallogr. Sect. D Struct. Biol.* **2009**, *65*, 148–155. https://doi.org/10.1107/s090744490804362x.

[S7] Dolomanov, O.V.; Bourhis, L.J.; Gildea, R.J.; Howard, J.A.K.; Puschmann, H. OLEX2: A complete structure solution, refinement and analysis program. *J. Appl. Cryst.* **2009**, *42*, 339–341, https://doi.org/10.1107/s0021889808042726.

[S8] Taylor, R.; Macrae, C.F. Rules governing the crystal packing of mono- and dialcohols. *Acta Cryst.* **2001**, *B57*, 815–827.

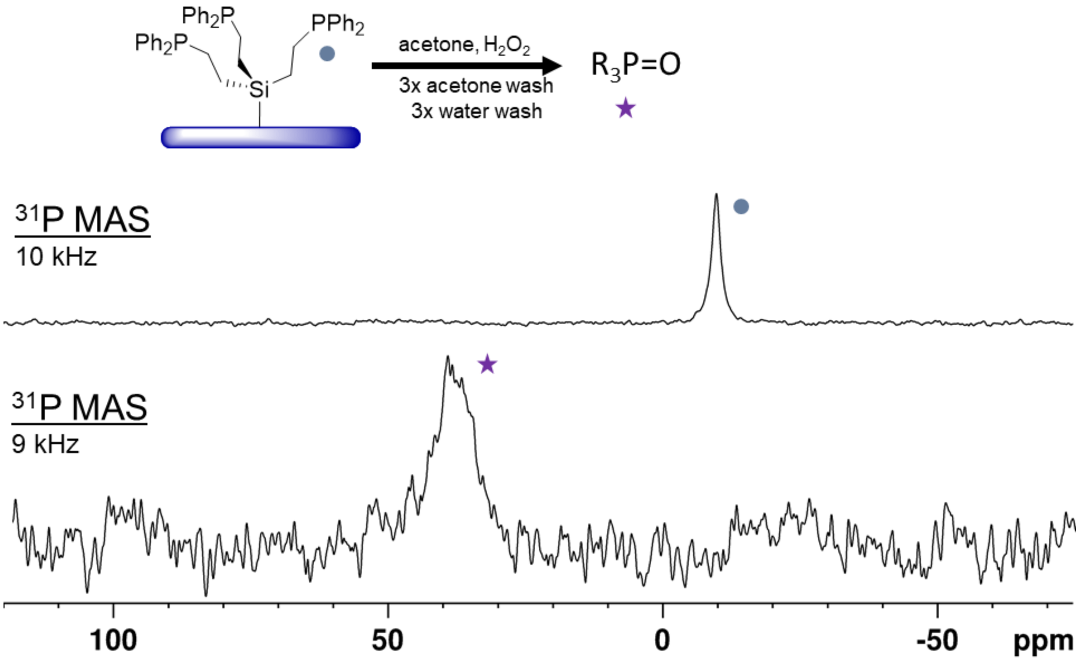

Figure S13. $^{31}P\{^{1}H\}$ MAS NMR spectra of **3i** (top) and of **3i** after oxidation with hydrogen peroxide (bottom) at the indicated rotational speeds.
